# Multidisciplinary Management of a Fused Tooth: A Case Report

**DOI:** 10.1155/2013/634052

**Published:** 2013-12-11

**Authors:** Derya Demircioglu Guler, Emine Sen Tunc, Nursel Arici, Nilüfer Ozkan

**Affiliations:** ^1^Department of Pediatric Dentistry, Faculty of Dentistry, Ondokuz Mayis University, Kurupelit, 55139 Samsun, Turkey; ^2^Department of Orthodontics, Faculty of Dentistry, Ondokuz Mayis University, Kurupelit, 55139 Samsun, Turkey; ^3^Department of Oral and Maxillofacial Surgery, Faculty of Dentistry, Ondokuz Mayis University, Kurupelit, 55139 Samsun, Turkey

## Abstract

*Objective*. Fusion is a dental anomaly that arises through the union of two adjacent teeth. The case report presents multidisciplinary management of a fused maxillary anterior tooth. *Case Report*. A 10-year-old boy was referred to the pediatric dental clinic with the chief complaint of a large upper anterior tooth. Intraoral and radiographic examinations indicated fusion between the permanent maxillary right central incisor and a supernumerary tooth. According to the treatment plan, the fused tooth was sectioned, and the mesial portion was removed. The remaining tooth section was restored with composite resin, and the diastema between the central incisors was closed with orthodontic treatment. After an 18-month followup period, the tooth showed no sign of pathosis. *Conclusion*. The technique described here offers a simple and effective method for restoring a fused tooth that reestablishes function, shape, and esthetics.

## 1. Introduction

Tooth fusion is a rare developmental anomaly that stems from the embryogenic union of two teeth originating from two or more tooth germs [1, 2]. Although the exact etiology of fusion is unclear, pressure or physical force producing close contact between two developing tooth buds has been reported as a possible cause, and trauma, genetic and environmental factors have also been implicated as contributing factors [3–6]. Moreover, fused teeth may form part of syndromes such as achondrodysplasia, chondroectodermal dysplasia, focal dermal hypoplasia, and osteopetrosis [7–11].

The literature suggests fusion has a higher incidence in deciduous dentition (0.5%–2.5%) than in permanent dentition (0.1%–1.0%) [7, 12, 13]. Fused teeth are found predominantly in the anterior region, with incisors and canines the most frequently affected [14, 15]. These anomalies may be bilateral or unilateral [14, 16]. The incidence of fusion does not vary by sex [7, 12]. Fused teeth may occur in both the maxilla and mandible, but they are more frequently present in the mandible [17].

The majority of fused teeth show an anomalous broad crown and two distinct root canals. Clinically, the crowns appear melded together, with a small groove between the mesial and distal parts. Fused teeth may be characterized by one pulp chamber divided into two root canals, two independent endodontic systems, or one common pulp canal [5, 15, 18].

This case report presents the surgical separation and orthodontic treatment of a maxillary central incisor fused with a supernumerary tooth.

## 2. Case Report

A 10-year-old boy was referred to the paediatric dental clinic with the chief complaint of the unpleasant appearance of a large anterior tooth. His medical history was noncontributory. There was no family history of dental anomalies, and there was no previous trauma to the teeth or jaws. The patient was a single child of a nonconsanguineous marriage.

**Figure 1:**
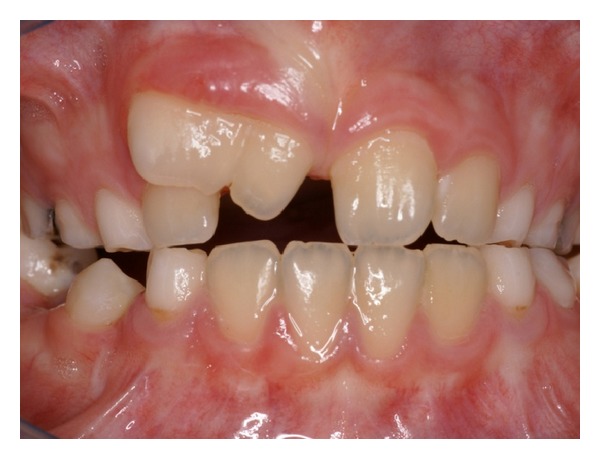
Clinical appearance of fused tooth.

Intraoral examination revealed a large maxillary right central incisor, with a small groove observed on the labial and palatal aspects of the crown (Figure 1). Response to pulp testing was normal, no caries were detected, and there was no significant periodontal pocketing. The patient was in mixed dentition and had crowding in the anterior region due to a lack of space. The total number of teeth was normal.

**Figure 2:**
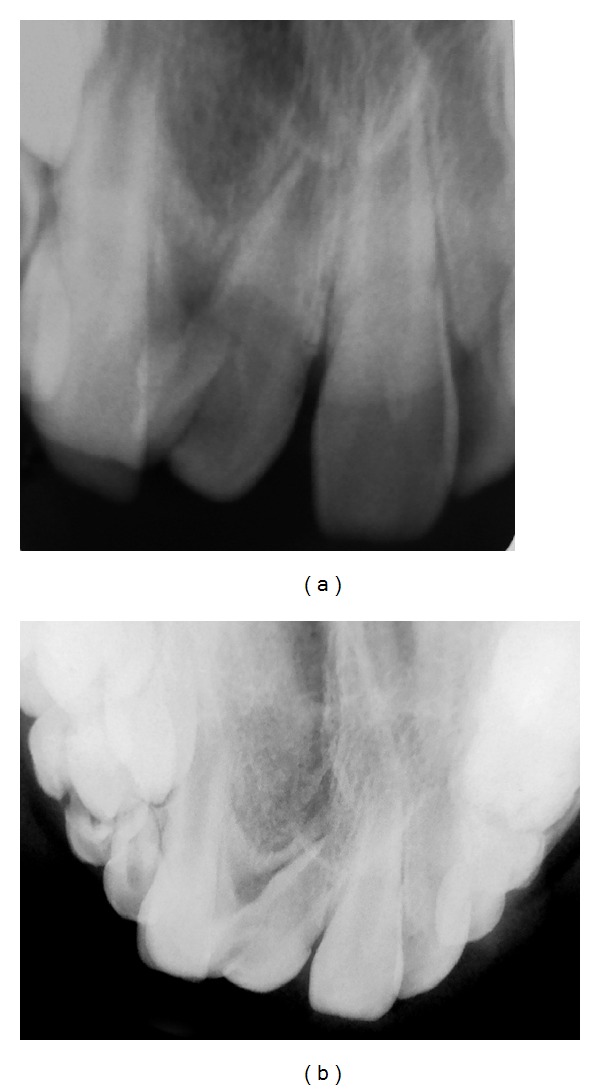
Radiographic appearance of fused tooth.

Radiographic examination revealed fusion of the maxillary right permanent central incisor with a supernumerary tooth. According to the radiographs, the fused tooth had two distinct roots, with no connection between the pulp chambers and canals. No periapical radiolucency associated with these teeth was detected (Figures 2(a) and 2(b)).

An orthodontic examination was conducted, and an initial treatment plan was developed that aimed to separate the fused supernumerary tooth and then correct the arch discrepancy with orthodontic treatment. The treatment objectives and alternatives were explained to the patient and his parents who provided their written consent prior to treatment.

**Figure 3:**
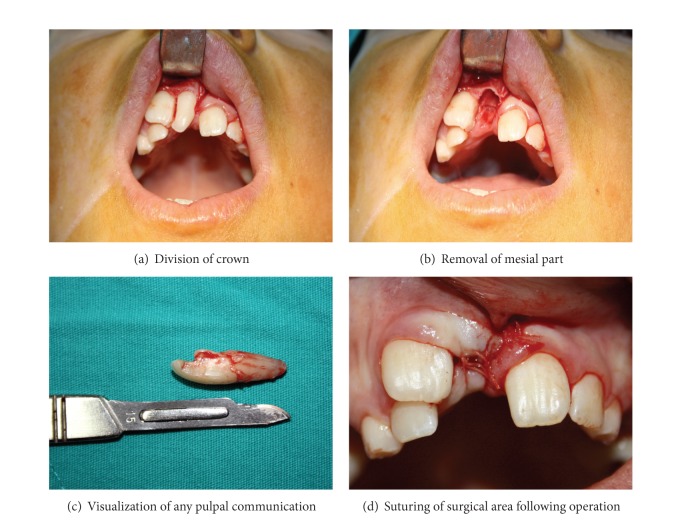
Surgical procedures.

The teeth were anaesthetized (Ultracain D-S, Istanbul, Turkey), and buccal and palatal flaps were raised. Some bones were removed below the buccal groove in order to locate the point at which the two roots separated. The crown was sectioned with a diamond bur (Midwest, Dentsply, IL, USA), and the mesial part of the tooth was removed (Figures 3(a) and 3(b)). The root surfaces were examined, and no pulpal association was detected (Figure 3(c)). The remaining distal tooth was restored with composite resin (Gradia Direct, GC Corporation, Tokyo, Japan), and the flaps were sutured (Figure 3(d)). Following surgery, the patient was prescribed chlorhexidine mouthwash (Orasept, Biofarma İlaç San. ve Tic. A.Ş, İstanbul, Turkey). Sutures were removed one week postoperatively.

**Figure 4:**
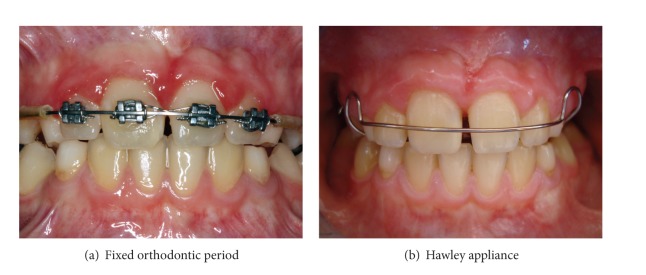
Orthodontic therapy.

Fixed orthodontic treatment was then agreed in order to correct the position of the central tooth, and routine orthodontic records (plaster model, photographs, and radiographs) were obtained. Clinical examination and analysis of the records showed the maxillary right central tooth to be infrapositioned. Cephalometric analysis showed the upper and lower jaws to be in normal position in the sagittal plane in relation to the base of the cranium, but the right upper central tooth was found to be protruding slightly due to the fusion. In order to eliminate the malocclusion, a fixed orthodontic appliance was planned using 0.022 inch Gemini MBT metal brackets (3 M Unitek, USA). Following placement of the maxillary incisor brackets, an arch wire (16 Heat Active Nickel-Titanium, 3 M Unitek) was connected to the bracket slots to correct the position of the maxillary right central tooth (Figure 4(a)).

At the fixed appliance adaptation appointment, the patient was instructed on oral hygiene and prohibited foods and was called back for routine orthodontic controls once a month. When the maxillary right central incisor had moved into the correct position in the arch, 0.019′′ × 0.025′′ Heat Active Nickel-Titanium and 0.019′′ × 0.025′′ stainless steel arch wires were attached to correct the midline diastema. At the end of a 13-month followup period, orthodontic treatment using fixed mechanics was found to have achieved adequate positioning of the central teeth. Following fixed orthodontic treatment, a Hawley appliance was constructed for retention (Figure 4(b)).

**Figure 5:**
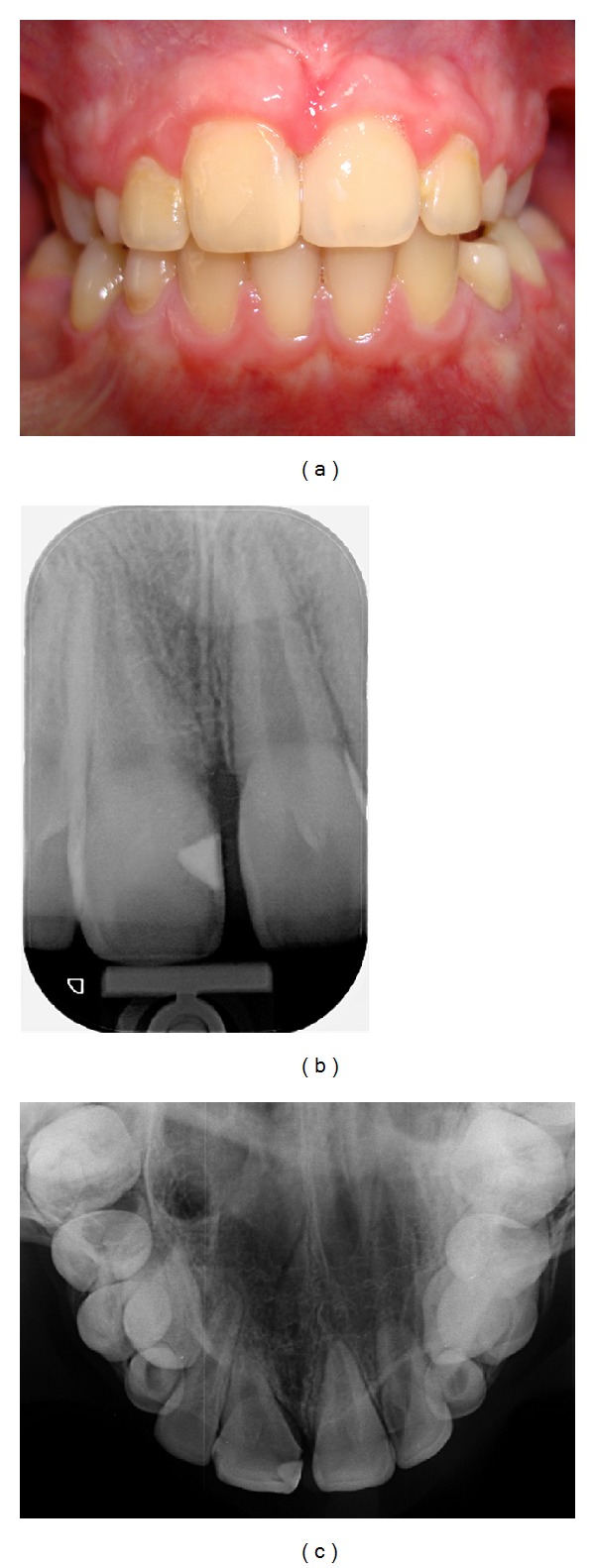
Clinical and radiographic appearance at 18-month followup.

Once orthodontic treatment had been completed, the maxillary central teeth were reshaped with composite resin (Gradia Direct, GC Corporation, Tokyo, Japan) for aesthetic reasons. Teeth were checked for possible complications once every three months. After an 18-month followup period, the maxillary right central tooth showed no sign of periapical pathosis (Figures 5(a) and 5(b)) and responded positively to electric pulp testing. Followup examinations indicated that treatment had successfully restored both esthetics and function. Probing revealed no periodontal pocketing around the central incisor, and there was good attachment; however, long-term followup is required.

## 3. Discussion

Fused teeth may lead to serious esthetic problems and malocclusions, especially when supernumerary elements are involved. Owing to the abnormal shape and size of crown(s) and root(s) as well as misalignment, treatment usually requires a multidisciplinary approach to address both endodontic and esthetic issues [9, 19, 20].

Depending on factors such as location of the connecting area, root-development stage, and patient age, treatment of fused teeth may vary [15]. One option involves extraction of the fused tooth after completion of root-canal therapy, removal of the unwanted part, and reimplantation of the tooth into its original site [21, 22]. However, because the root surface of the reimplanted tooth lacks a periodontal membrane, ankylosis may be expected [23]. In the present case, based on the location of the connecting area, the root-development stage, and the patient's age, a more conservative treatment option was selected. Because the fused tooth possessed two separate roots and canals, extraction of the mesial part of the tooth was chosen to solve the esthetic problems and obtain space for alignment. Although endodontic treatment may be necessary even when no pulpal communication is visualized between two fused teeth, in the case presented here, no pulpal exposure was observed following separation, and no endodontic therapy was required. Difficulties in the observation of the surgical area require clinicians to check carefully for any possible pulpal exposure, and detailed radiographic followup examinations are essential for avoiding late-term complications.

In the present case, following hemisection of the crown, the remaining part was restored with traditional composite resin. Some case reports have described postoperative complications such as hypersensitivity, reversible pulpitis, and external root resorption [22]. In this case, no pathological findings were observed radiographically, and the tooth responded positively to electric pulp testing at the one-year recall. Followup indicated that treatment had successfully restored both esthetics and function. No periodontal pocketing was found around the central incisor upon probing, and the attachment was good. Most importantly, the patient was pleased with the esthetic outcome of treatment.

## 4. Conclusion

Gemination and fusion are frequently responsible for periodontal, endodontic, orthodontic, and esthetic problems. The excellent esthetic and functional results obtained in the case presented here are attributable to scientific knowledge, broad experience, and team work, where individual experts in specialized fields contributed to the selection of the most suitable treatment possible.
